# Delineation of the HPV11E6 and HPV18E6 Pathways in Initiating Cellular Transformation

**DOI:** 10.3389/fonc.2017.00258

**Published:** 2017-11-01

**Authors:** Lamech M. Mwapagha, Nicki Tiffin, M. Iqbal Parker

**Affiliations:** ^1^Faculty of Health Sciences, Division of Medical Biochemistry and Structural Biology, Institute of Infectious Disease and Molecular Medicine, University of Cape Town, Cape Town, South Africa; ^2^International Centre for Genetic Engineering and Biotechnology, Cape Town Component, Cape Town, South Africa; ^3^Centre for Infectious Disease Epidemiology and Research, School of Public Health and Family Medicine, University of Cape Town, Cape Town, South Africa

**Keywords:** cellular transformation, human papillomavirus, E6 gene, differentially expressed genes, DNA microarray analysis

## Abstract

Although high-risk human papillomaviruses (HPVs) are the major risk factors for cervical cancer they have been associated with several other cancers, such as head and neck and oral cancers. Since integration of low-risk HPV11 DNA has been demonstrated in esophageal tumor genomes, this study compared the effects of low-risk HPV11E6 and high-risk HPV18E6 on cellular gene expression. The HPV11E6 and HPV18E6 genes were cloned into an adenoviral vector and expressed in human keratinocytes (HaCaT) in order to investigate early events and to eliminate possible artifacts introduced by selective survival of fast growing cells in stable transfection experiments. HPV11E6 had very little effect on p21 and p53 gene expression, while HPV18E6 resulted in a marked reduction in both these proteins. Both HPV11E6 and HPV18E6 enabled growth of colonies in soft agar, but the level of colony formation was higher in HPV18E6 infected cells. DNA microarray analysis identified significantly differentially regulated genes involved in the cellular transformation signaling pathways. These findings suggest that HPV11E6 and HPV18E6 are important in initiating cellular transformation via deregulation of signaling pathways such as PI3K/AKT and pathways that are directly involved in DNA damage repair, cell survival, and cell proliferation. This study shows that the low-risk HPV11E6 may have similar effects as the high-risk HPV18E6 during the initial stages of infection, but at a much reduced level.

## Introduction

Human papillomaviruses (HPV) are implicated in a number of human squamous cell carcinomas, including skin, cervix, anogenital, and upper respiratory tract cancers (1). To date nearly 150 different HPV types have been identified, of which 120 have been fully sequenced. Some of these (high-risk HPV 16 and 18) have been associated with cancers, whereas others (HPV 6, 11, and 33) give rise to warts and benign lesions and are considered low-risk (2). Despite the substantial financial burden imposed on the health systems by the low-risk HPV types, most of the basic and clinical research have focused on the high-risk HPV types (3). The molecular mechanisms by which the low-risk HPV E6 protein may contribute to malignant progression are not fully understood (4–6).

High-risk HPV E6 functions as an oncoprotein, with its primary activity being the targeting p53 for degradation *via* the cellular ubiquitin ligase E6-AP pathway (7–16). The degradation of p53 in turn compromises the integrity of the cellular genome resulting in increased DNA damage, chromosomal instability, increased cell proliferation, and subsequent tumorigenesis (17–19). As a consequence of the E6-mediated degradation of p53, p21 gene expression is also inhibited. Although there are structural differences between the E6 from HPV11 and HPV18, novel functions have been identified for the low-risk E6 that were previously observed for high-risk HPV proteins and may reflect common pathways utilized by both types of viruses during their productive life cycles (20–22).

Various cell types, such as human prostatic, cervical, and ovarian epithelial cells, have been used to investigate the underlying mechanisms of HPV-induced malignant transformation (23, 24). Although HaCaT cells represent a spontaneously immortalized human keratinocyte cell line expressing mutant p53, they still maintain a non-tumorigenic phenotype consistent with the growth suppressive properties of the wild type p53. Since HaCaT cells require specific genetic alterations for tumorigenic conversion that do not occur spontaneously under standard culture conditions (25), these cells also offer a suitable model to study regulatory mechanisms in the differentiation of human epidermal cells and provide a valuable model system to study the role of oncogenes and other factors in the process of malignant transformation (24–28).

This study compared some of the biological changes in the HPVE6-infected HaCaT cells following transient expression of low-risk HPV11E6 or high-risk HPV18E6 genes introduced into the cells *via* a recombinant adenovirus expression vector. Previous studies have used stably transfected cells where clonal selection after several generations of subculture could result in the domination and eventual selection of only the most rapidly growing cells in the population (2, 29–32). In this study, we utilized an adenoviral expression vector to transiently express either HPV11E6 or HPV18E6 genes, with subsequent gene expression analysis being done without sub-culturing of the cells. This study, therefore, provides an understanding of the mechanisms involved in the very early stages of cellular transformation and also provides a better understanding of the underlying mechanisms of low-risk HPV in this process.

## Results

### Transient Infection of HaCaT Cells

**Figure 1:**
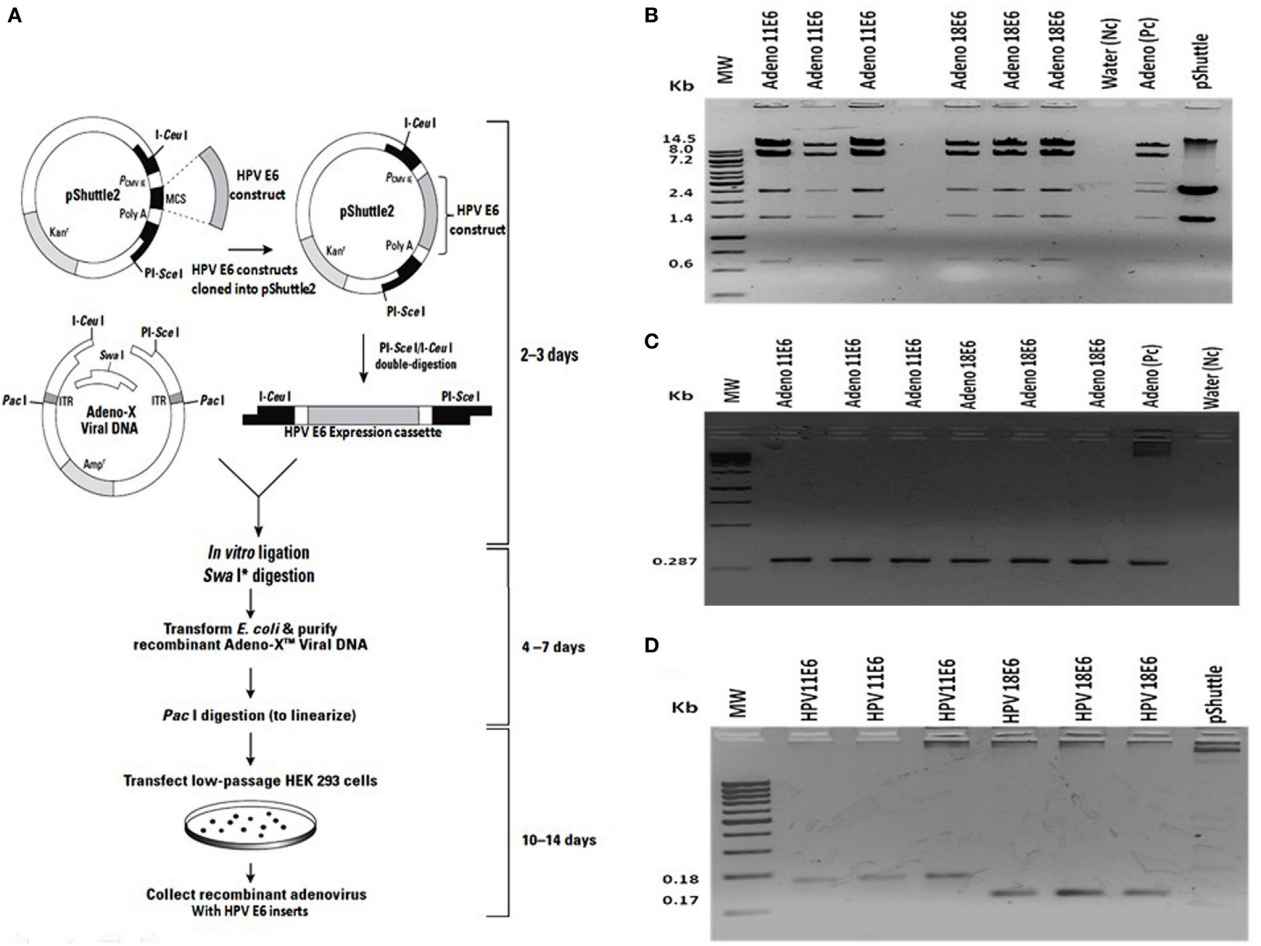
Construction of recombinant adeno-E6 vectors. **(A)** The human papillomavirus (HPV) genes were removed from pcDNA3.1 and cloned into Adeno X as outlined (Image modified from Clontech Labs., Adeno-X™ Expression System 1). **(B)** Verification of cloning of the HPVE6 expression cassette by digestion of the constructs, an Adeno-X positive control (Pc) and the pShuttle2 vector negative control (Nc) with XhoI. **(C)** Verification of recombinant Adeno-HPVE6 constructs by PCR with the Adeno-X forward and reverse PCR primers that specifically amplifies a 287-bp sequence spanning the I*Ceu*I ligation site. **(D)** Verification of HPV E6 constructs using HPV11E6 and HPV18E6 specific primers with pShuttle as a Nc. In each case, three different clones of each construct were selected for analysis **(B–D)**.

**Figure 2:**
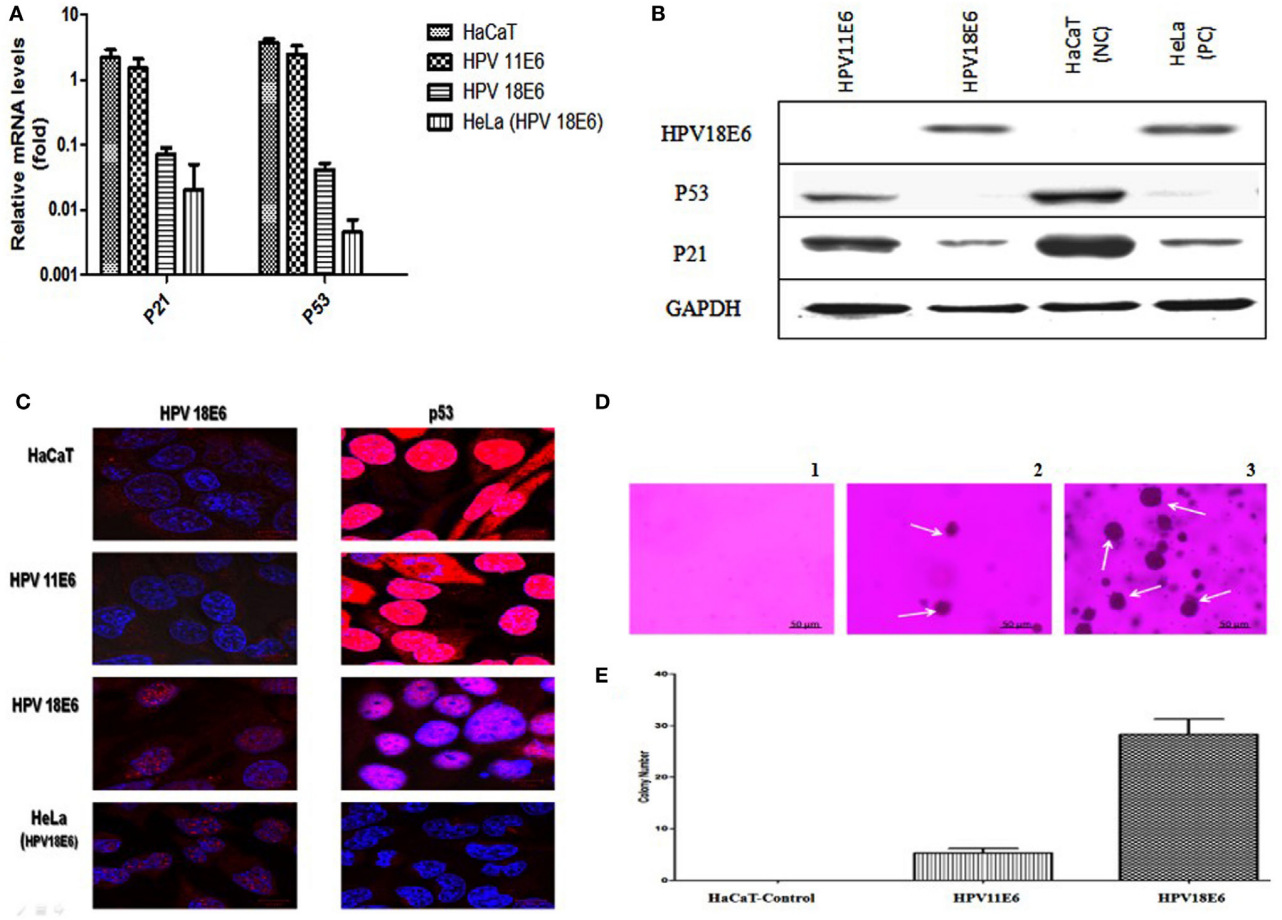
HPVE6 induces cellular transformation. **(A)** Quantitation of p21 and p53 mRNA in the adenovirus-HPVE6-infected cells as determined by qRT-PCR. A reduction in the mRNA levels of both genes in HPV18E6-infected HaCaT cells and the positive control (PC) HeLa cells was seen as opposed to the unchanged levels in the HPV11E6 infected cells. **(B)** Analysis of HPV18E6 (18 kDa), P21 (21 kDa), and P53 (53 kDa) protein levels by western blot analysis showed a marked reduction of both P53 and P21 in the HPV18E6 infected HaCaT cells but only a marginal decrease in the HPV11E6 cells. Uninfected HaCaT cells served as negative control (Nc) and HeLa cells are known to constitutively express the HPV18E6 protein served as PC. **(C)** The localization of HPV18E6 and P53 in HaCaT cells infected with HPV11E6 and HP18E6 was determined by confocal microscopy. In HPV11E6-expressing cells, p53 was localized primarily in the nucleus with some diffused staining in the cytoplasm, whereas in HPV18E6-expressing cells, p53 was observed at lower levels than either in the control or the HPV11E6 infected cells, with increased cytoplasmic staining. **(D,E)** Anchorage-independent cell growth as determined by colony formation in soft agar. Cells were plated at a density of 2.5 × 10^3^ cells/well and colony formation determined after 21 days as described in the Section “Materials and Methods.” Analysis of the colonies was done using the ImageJ software (33), with colonies larger than 150 μm in diameter being scored as positive. Graph shows mean number of colonies (**±**SE) obtained in three independent experiments, with each experiment performed in six replicate wells.

The HPVE6 genes were cloned into an adenoviral expression vector as outlined in Figure 1A and the inserts confirmed by PCR (Figure 1B) and DNA sequence analysis as shown in Figure S1. Recombinant Adeno-HPVE6 constructs were confirmed by PCR using Adeno-X forward and reverse PCR primers (Figure 1C), while the verification of HPV E6 constructs were done using HPV11E6 and HPV18E6 specific primers (Figure 1D). HaCaT cells were infected with either HPV11E6 or HPV18E6 constructs for 48 h and the levels of p21 and p53 mRNA and proteins determined by qRT-PCR and western blot analysis respectively. Expression of the p21 and p53 genes in cells expressing HPV11E6 were marginally reduced as opposed to cells expressing HPV18E6 where a marked reduction of both p21 and p53 mRNA and protein was observed (Figures 2A–C). These findings confirmed that p53 was degraded in cells expressing HPV18E6, leading to the inactivation of the p21 gene. The potential of HPV11E6 and HPV18E6 to induce degradation or relocalization of p53 was assessed by confocal microscopy. In HPV11E6 expressing cells, p53 was localized primarily in the nucleus with some diffused staining in the cytoplasm, whereas in HPV18E6 expressing cells, p53 was observed at lower levels than either in the control HaCaT cells or in the HPV11E6 expressing cells. This was accompanied by increased cytoplasmic staining, probably as p53 degradation products (Figure 2D). Control HaCaT cells did not produce any colonies in soft agar, while a few colonies were present in cells expressing HPV11E6 while a considerable number were present in cells expressing HPV18E6 (Figure 2E). These results confirm the ability of both HPVE6 types to induce cellular transformation, but with HPV18E6 having a much stronger effect.

#### HPV E6 Sequence Confirmation

In order to verify that the recombinant adenoviral construct contained a full-length copy of the HPV E6 gene, PCR amplification, and DNA sequence analysis was done using the HPV11E6 or HPV18E6 specific primers. A BLASTn search showed 100% nucleotide sequence identity to the HPV11E6 (Accession number: FN870475.1) and HPV18E6 (Accession number: EF422110.1) sequences on the NCBI database. The sequences were interpreted using the Chromas software version 2.01 (34) (Figure S1 in Supplementary Material).

#### E6-Induced Differential Gene Expression

**Figure 3:**
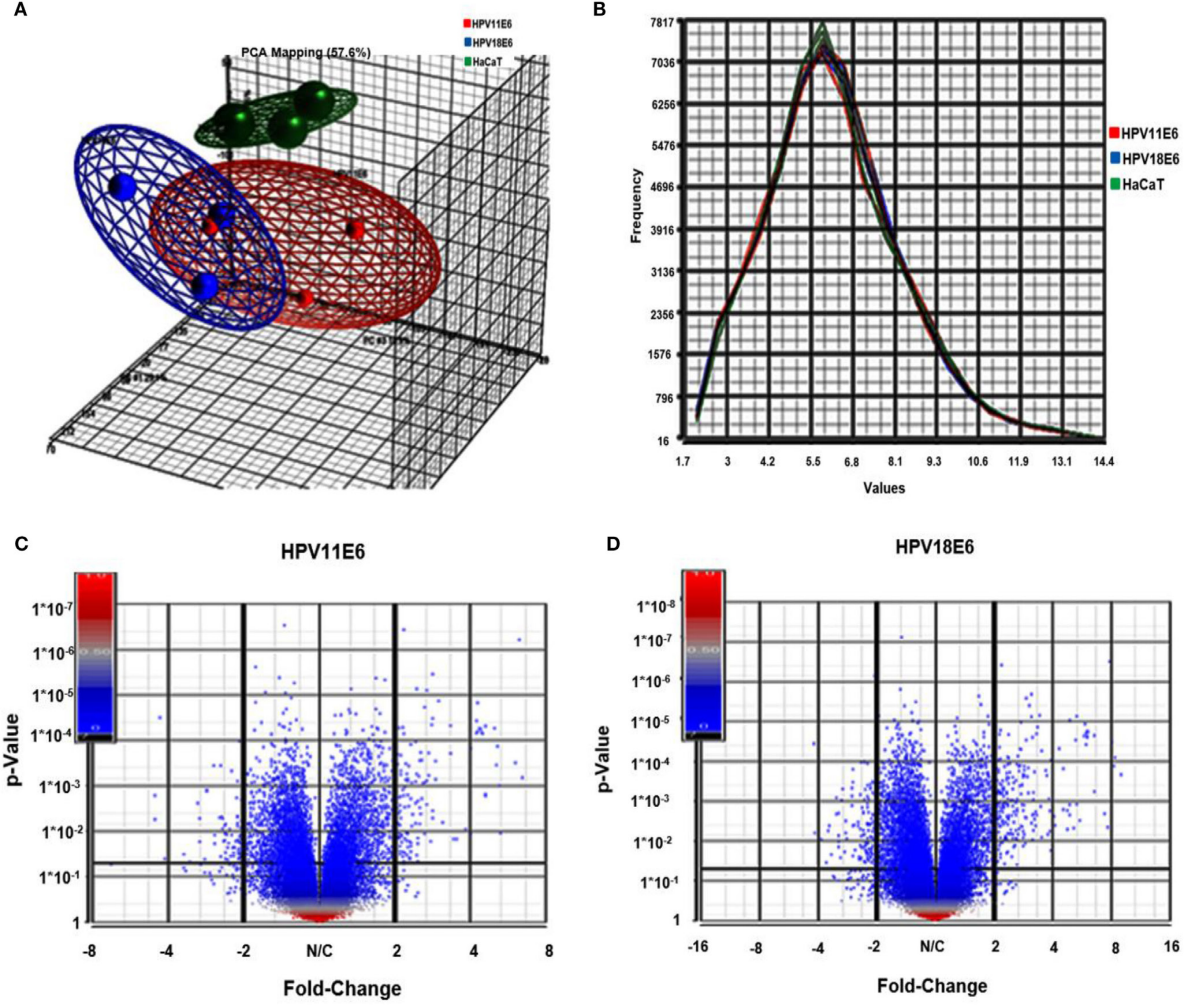
Analysis of microarray data in the infected HaCaT cells. **(A)** Principal component analysis (PCA) analysis plots (showing one dot for each of the triplicate samples) for the HaCaT control cells (green), the HPV11E6 (red), and the HPV18E6 (blue) cells. The HPV11E6 and the HPV18E6 cells had some similarity to each other and both were distinctly different from the control cell. **(B)** Line graph plots with one line for each of the samples with the intensity of the probes indicated on the *X*-axis and the frequency of the probe intensity on the *Y*-axis. In this dataset, all the samples follow the same distribution pattern indicating that there are no obvious outliers in the data. **(C,D)** Volcano plots of HaCaT cells expressing HPVE6 vs. HaCaT control cells with each dot representing a gene. The *X*-axis shows the fold change and the *Y*-axis the *p*-values. The up- and downregulated genes are on the right- and left-hand side, respectively.

**Figure 4:**
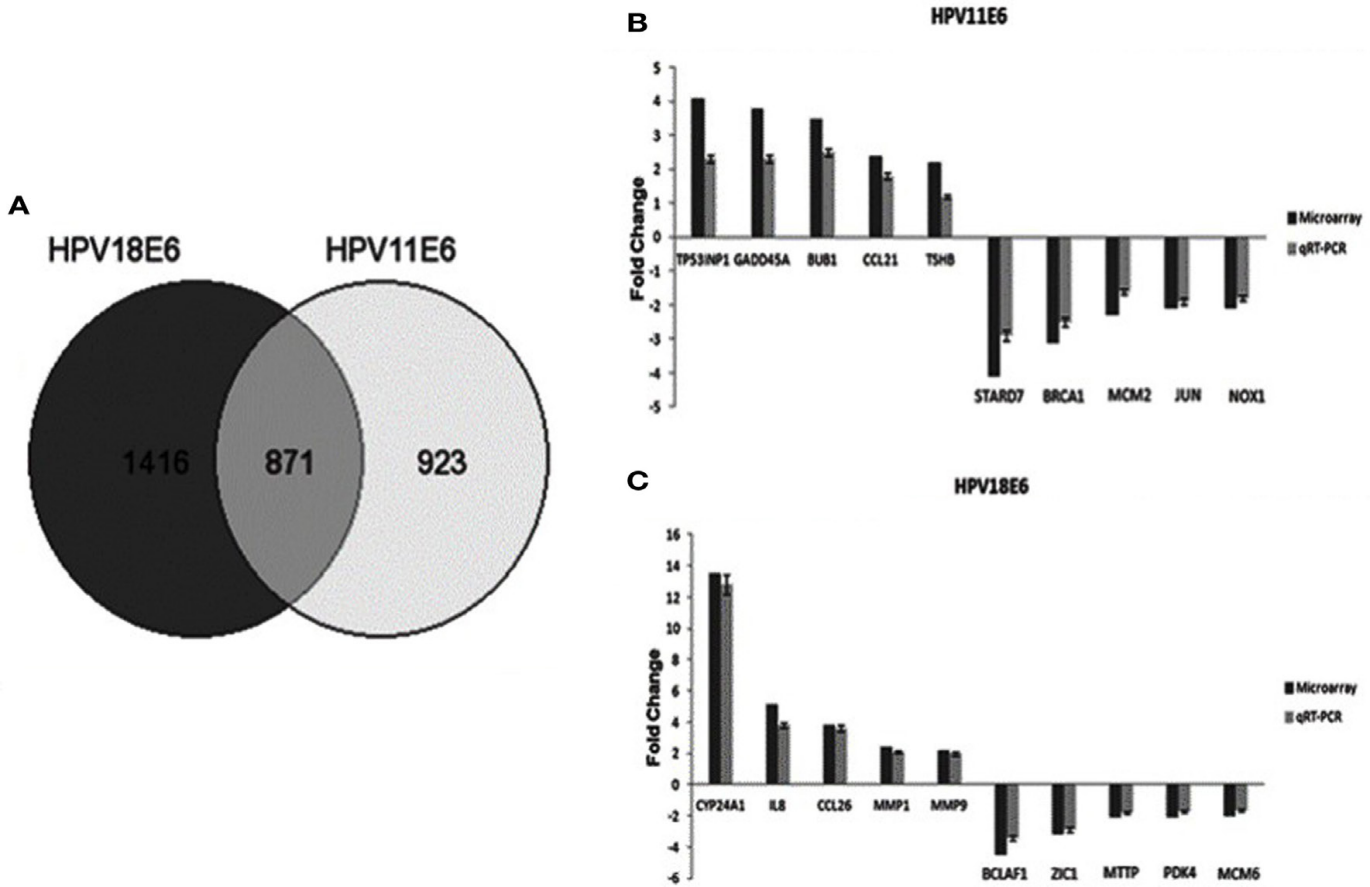
The effect of HPVE6 on cellular gene expression patterns. **(A)** A Venn diagram showing the number of significantly differentially expressed genes (DEGs) (both up- and downregulated). A total of 3,210 genes were significantly differentially expressed, of which 1,416 genes were uniquely deregulated in HPV18E6 cells, while 923 genes were uniquely deregulated in HPV11E6 cells and 871 genes were deregulated by both HPVs compared to the control HaCaT cells. **(B)** HPV11E6 and (C) HPV18E6, qRT-PCR validation of the microarray data showing the selected top 10 DEGs based on either the extent of up- or downregulation or on their known involvement in cellular transformation. The fold change shown was normalized to GAPDH mRNA levels and qRT-PCR figures represent the average of three replicates for each infection with the error bars indicating SD.

Cellular gene expression was determined by DNA microarray analysis after infection of HaCaT cells with either HPV11E6 or HPV18E6. The robust multi-array average (RMA) approach was used in the normalization of the raw data. Principal component analysis (PCA) revealed some overlap in the gene expression pattern between the two HPVE6 expressing cells but both were clearly distinct from the uninfected HaCaT control cells (Figure 3A). There were no obvious outliers in the data as shown in the probe intensity plots in Figure 3B. Volcano plots set at a fixed fold-change cutoff of ±2.0 with significance FDR of ≤0.05 generated a list of genes with a significant differential expression pattern for HPV11 and HPV18 (Figures 3C,D). A total of 3,210 genes were significantly differentially expressed, of which 1,416 genes were uniquely differentially regulated in HPV18E6 expressing cells, while 923 genes were uniquely differentially regulated in HPV11E6 expressing cells (Figures 4A) and 871 genes were differentially regulated by both HPVs. This result is consistent with the PCA result (Figure 3A) that showed that both HPV11E6 and HPV18E6 shared some common differentially regulated genes. Data generated from the microarray analysis were validated by qRT-PCR for the top 10 differentially expressed genes (DEGs) based on either the extent of up- or down regulation or on their known involvement in carcinogenesis (Tables 1–3). The qRT-PCR results showed that the trends in expression for the selected genes were consistent with the microarray data, verifying that the microarray data were accurate and reliable (Figures 4B,C).

**Table 1:** HPV11E6 Top 10 up- and downregulated gene based on either the extent of up- or downregulation or on their known involvement in cellular transformation.

Up-regulated			Down-regulated		
Gene symbol	Gene name	Fold change	Gene symbol	Gene name	Fold change
PLAT	Plasminogen activator, tissue	4.3	STARD7	StAR-related lipid transfer domain containing 7	−4.1
TNFSF10	Tumor necrosis factor (ligand) superfamily, member 10	4.2	BRCA1	Breast cancer 1, early onset	−3.1
TP53INP1	Tumor protein p53 inducible nuclear protein 1	4.1	OR2M7	Olfactory receptor, family 2, subfamily M, member 7	−3.0
GADD45A	Growth arrest and DNA-damage-inducible, alpha	3.8	IFI6	Interferon, alpha-inducible protein 6	−2.4
BUB1	Budding uninhibited by benzimidazoles 1	3.5	MCM2	Minichromosome Maintenance complex component 2	−2.3
BHLHE41	Basic helix-loop-helix family, member E41	3.4	TMEM27	Transmembrane protein 27	−2.2
FBP1	Fructose-1,6-bisphosphatase 1	3.3	NOX1	NADPH oxidase 1	−2.1
LXN	Latexin	3.1	JUN	Jun proto-oncogene	−2.1
EPPK1	Epiplakin 1	3.0	SNAR-E	Small ILF3/NF90-associated RNA E	−2.1
PIGR	Polymeric immunoglobulin receptor	2.9	CXCL11	Chemokine (C-X-C motif) ligand 11	−2.1

**Table 2:** HPV18E6 top 10 up- and downregulated genes based on either the extent of up- or downregulation or on their known involvement in carcinogenesis.

Up-regulated			Down-regulated		
Gene symbol	Gene name	Fold change	Gene symbol	Gene name	Fold change
CEACAM5	Carcinoembryonic antigen-related cell adhesion molecule 5	22.7	ANKRD1	Ankyrin repeat domain 1 (cardiac muscle)	−4.8
CYP24A1	Cytochrome P450, family 24, subfamily A, polypeptide 1	13.5	BCLAF1	BCL2-associated transcription factor 1	−4.5
MMP12	Matrix metalloproteinase 12	11.7	FST	Follistatin	−4.0
GNE	Glucosamine (UDP-N-acetyl)-2-epimerase/N-acetylmannosamine kinase	10.2	TRAJ59	T cell receptor alpha joining 59 (non-functional)	−4.0
S100A7	S100 calcium binding protein A7	10.0	PTHLH	Parathyroid hormone-like hormone	−3.9
PNLIPRP3	Pancreatic lipase-related protein 3	9.9	IFI44L	Interferon-induced protein 44-like	−3.7
FABP4	Fatty acid binding protein 4, adipocyte	9.6	THBS1	Thrombospondin 1	−3.3
SERPINB4	Serpin peptidase inhibitor, clade b (ovalbumin), member 4	9.0	ODC1	Ornithine decarboxylase 1	−3.2
AKR1C2	Aldo-keto reductase family 1, member C2	8.4	ZIC1	Zinc finger of the cerebellum Family member 1	−3.2
TRIM31	Tripartite motif containing 31	8.4	TGFB2	Transforming growth factor, beta 2	−3.1

**Table 3:** HPV11E6 and HPV18E6 shared Top 10 up- and down-regulated genes based on either the extent of up- or downregulation or on their known involvement in cellular transformation.

Up-regulated			Down-regulated		
Gene symbol	Gene name	Fold change	Gene symbol	Gene name	Fold change
MIR4461	MicroRNA 4461	3.2	CEACAM 5	Carcinoembryonic antigen related cell adhesion molecule 5	−4.2
TRAJ59	T-cell receptor alpha joining 59	3.0	CYP1A1	Cytochrome P450, subfamily I	−2.6
PTHLH	Parathyroid hormone-like hormone	2.6	BCL6	B-cell CLL/lymphoma 6	−2.4
FST	Follistatin	2.6	MMP12	Matrix metalloproteinase 12	−2.3
KPNA1	Karyopherin subunit alpha 1	2.2	STOM	Stomatin	−2.3
SERPINB2	Serpin family B member 2	2.1	SNORD32B	Small nucleolar RNA, C/D box 32B	−2.3
INHBA	Inhibin beta A subunit	2.0	DMBT1	Deleted in malignant brain tumors 1	−2.2
MIR4481	MicroRNA 4481	2.0	LOXL4	Lysyl oxidase-like protein 4	−2.2
RN5S191	RNA, 5 S Ribosomal 191	2.0	DUOX2	Dual oxidase 2	−2.2
BTC	Betacellulin	2.0	CYP24A1	Cytochrome P450 family 24 subfamily a member 1	−2.0

#### Gene Ontology (GO) Enrichment Analysis

**Figure 5:**
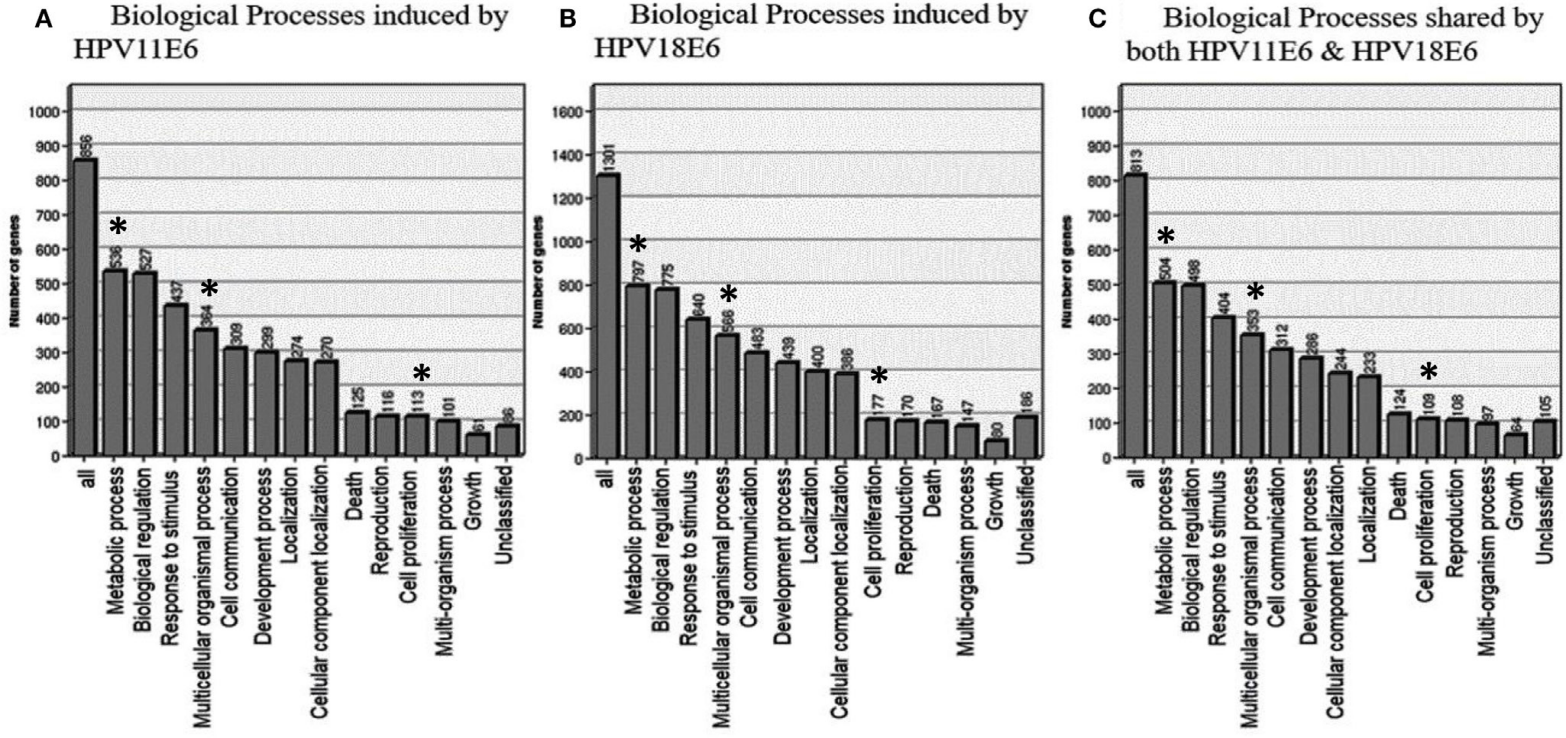
GOTree machine biological process analysis: graphical representation of enriched biological processes of differentially expressed genes (DEGs) following the expression of **(A)** HPV11E6, **(B)** HPV18E6, and **(C)** processes shared by both HPV11E6 and HPV18E6. HaCaT cells infected with HPV11E6 or HPV18E6 altered numerous unique biological processes, with 536 and 797 genes, respectively, being involved in the metabolic processes. While 504 DEGs shared by both HPV11E6 and HPV18E6 were also associated with the metabolic processes. The metabolic process is the most significantly enriched gene ontology (GO) function in the biological processes having the highest number of DEGs affected by HPVE6. Asterisks (*) depict the number of DEGs regulating the common GO processes that are associated with hallmarks of cancer following infection by HPVE6.

To determine the relevance of the DEGs, the GOTree machine (GOTM) was utilized to identify biological processes that were enriched in the set of 3,210 genes (Figure 4A) in the HaCaT-11E6 and HaCaT-18E6 cells. DEGs induced by HPV18E6 can be seen to be involved in a variety of functions ranging from metabolism, response to stress, cell proliferation, protein binding, cell cycle control, and cell communication. Importantly, these DEGs are also involved in cell shape and cytoskeletal re-organization pathways known to be altered during malignant transformation. HaCaT cells expressing HPV11E6 and HPV18E6 are shown here to alter numerous unique biological processes, with 536 and 797 genes, respectively, being involved in the metabolic processes (Figures 5A,B). In total, 504 DEGs were shared by both HPV11E6 and HPV18E6 (Figure 5C) that were also associated with metabolic processes. The extensive differential regulation of the metabolic process genes by HPVE6 was anticipated since tumors are known to be metabolically hyperactive and develop a highly specialized metabolic profile, while the regulation of important functions such as cell cycle, cell communication and cell signaling suggested an elevated cell proliferation capacity. Since these DEGs controlled key functions fitting the cancer hallmarks, the next step was to map these genes to known pathways so as to gain more insight into their underlying biological effects.

#### Signaling Pathway Analysis

**Figure 6:**
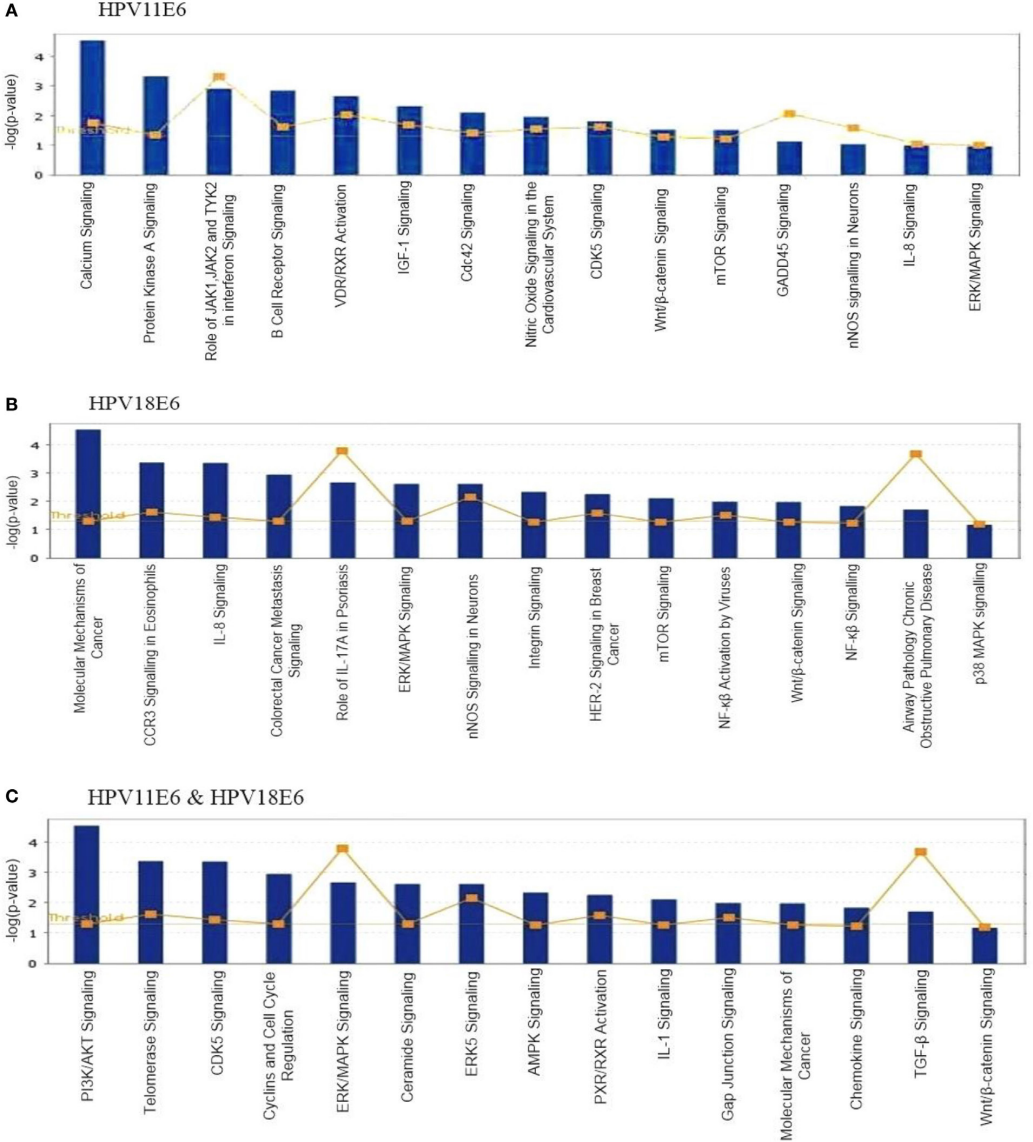
Ingenuity™ pathway analysis (IPA) canonical pathway analysis. Top pathways in which significant differentially expressed genes (DEGs) were enriched, following the expression of **(A)** HPV11E6; **(B)** HPV18E6; **(C)** HPV11E6, and HPV18E6 in HaCaT cells. Calcium signaling was the top significantly enriched pathway induced by HPV11E6 while HPV18E6 induced the molecular mechanisms of cancer as the top deregulated canonical pathway. The DEGs shared by both HPV11E6 and HPV18E6 induced PI3K/AKT signaling and telomerase signaling as the top most deregulated pathways. Bars represent -log (*p*-value) for disproportionate representation of affected genes in the selected pathway, the yellow boxed line represents the ratio of affected genes to the total number of genes in a pathway.

**Figure 7:**
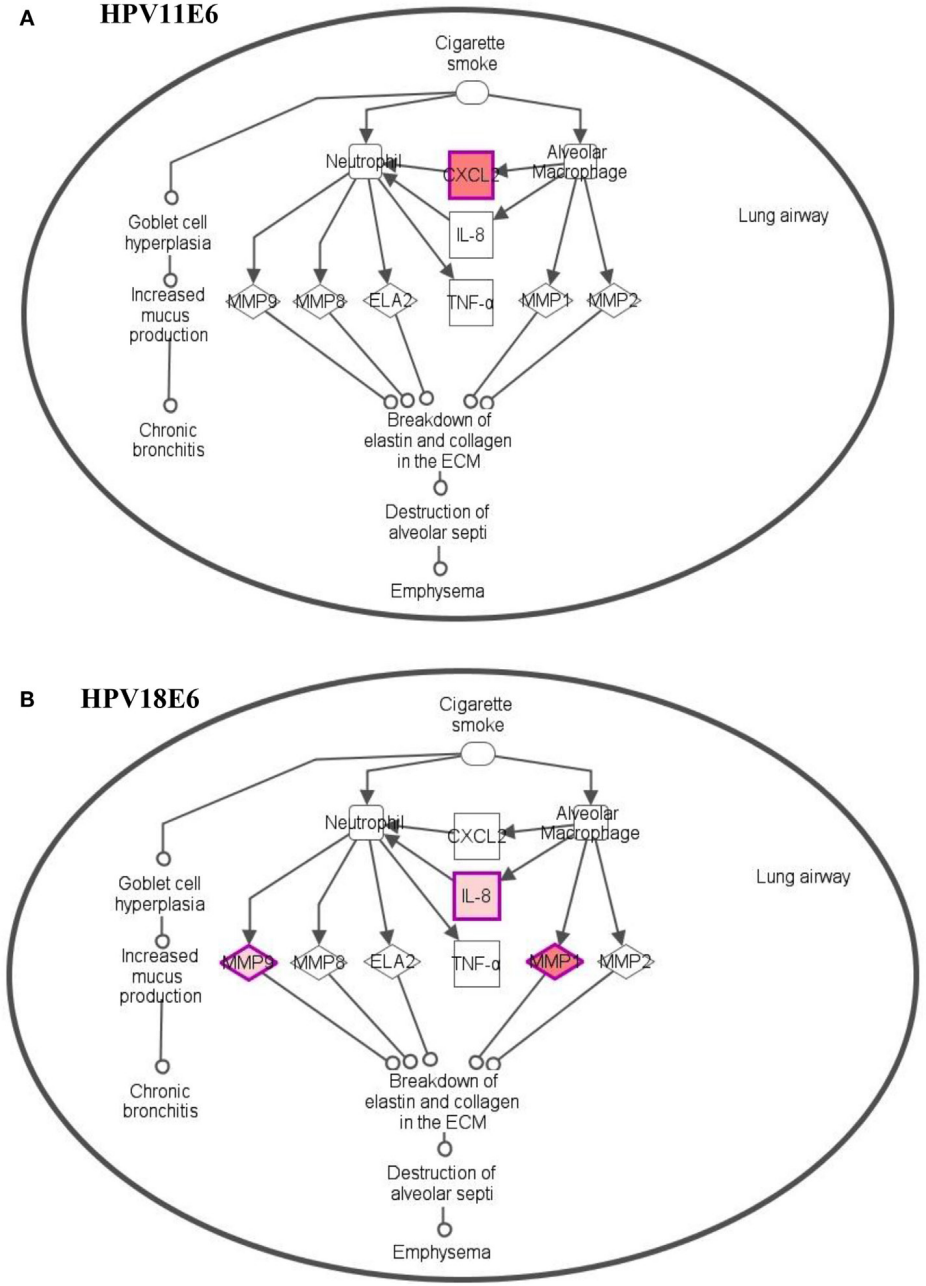
Differentially expressed genes induced in the chronic obstructive pulmonary disease canonical pathway. **(A)** HPV11E6 induced the upregulation of CXCL2 gene only, while HPV18E6 induced the upregulation of IL8, MMP1, and MMP9 genes **(B)**. Red shading indicates genes that are upregulated while the unshaded genes were not identified as differentially expressed by HPVE6 but were rather integrated into the computationally generated pathway on the basis of the evidence stored in the IPA knowledge memory indicating a relevance to this pathway.

To analyze the pathways deregulated at the early stages of HPVE6 gene expression we utilized the ingenuity™ pathway analysis (IPA) software. Calcium signaling, which is associated with enhanced cell proliferation and impaired apoptosis, emerged as the top significantly enriched pathway induced by HPV11E6 but not by HPV18E6. Other pathways of importance that were also differentially regulated were the JNK, mTOR, and GADD45 signaling pathways that are known to be altered during viral infections and with growth and development (Figure 6A). The cancer pathway, and the integrin signaling pathway that is associated with metastasis were the top differentially regulated canonical pathways induced by HPV18E6 (Figure 6B). The DEGs shared by both HPV11E6 and HPV18E6 included PI3K/AKT signaling, telomerase signaling, and cell cycle signaling as the top most deregulated pathways (Figure 6C). These pathways have previously been associated with DNA damage and cell proliferation (35, 36). The chronic obstructive pulmonary disease (COPD) canonical pathway was also one of the relevant pathways that was deregulated by HPV18E6 (Figure 7). Of significance is the upregulation of IL8, MMP1, and MMP9 that all have been shown to work synergistically in inducing cellular transformation (37–45).

## Discussion

While considerable work has been done on the mechanism(s) of action and the effects of high-risk HPVE6 on cellular gene expression patterns, not much is known about the function(s) of low-risk HPVE6 and the possible role that they may play in the process of cellular transformation. The goal of this study was to do a comparative study of the underlying mechanisms of the high and low-risk HPVs during the very early stages of cellular transformation. The approach used in this study was to transiently infect the HaCaT cell line with an adenoviral vector containing either the HPV11E6 or HPV18E6 genes and to investigate early changes in cellular gene expression patterns by DNA microarray analysis.

HaCaT cells expressing HPV11E6 showed a slight decrease in the expression of p21 and p53 in contrast to those infected with HPV18E6, where these proteins were not detectable. These findings demonstrate that cells expressing HPV18E6 efficiently degraded p53, leading to the inactivation of the p21and the inability to protect against cellular transformation (46). This finding is consistent with other studies that have shown that p21 gene transcription is inhibited by HPVE6-mediated p53 degradation and that its reduced expression is also observed in invasive squamous cell carcinomas (18, 47, 48).

The continued expression of p53 in HPV11E6 expressing cells is due to the fact that HPV11E6 binds p53 with low affinity, resulting in a reduced ability to induce cellular transformation (21, 23). These results are consistent with studies of Stewart et al. (49) who showed that high-risk HPV18E6 but not low-risk HPV11E6 accumulate predominantly in the nucleus where it binds p53 and is then shuttled from the nucleus to the cytoplasm where the p53 is degraded. The reduced transformation ability of HPV11E6 is shown by the induction of very few and very tiny colonies in soft agar compared to cells expressing the high-risk HPV18E6. The reduced ability HPV11E6-infected cells to form colonies in soft agar may be attributed to the lower levels of induction of signaling pathways in involved in cellular transformation (7, 21).

The cellular gene expression pattern in the HPVE6 expressing cells differed significantly from that of the control HaCaT cells, but there was some similarity between the HPV11E6- and HPV18E6-infected cells as can be seen from the overlap of genes in the PCA. This finding is consistent with previous studies on stably transfected cells that showed some similarity in the high-risk HPV18E6- and low-risk HPV11E6-induced genes (7, 21). It is also noteworthy that the metabolic process gene set was most significantly enriched by both HPV11E6 and HPV18E6. This could be ascribed to increased energy metabolism required for increased cell division and HPV replication (50). Importantly, the DEGs in HPV18E6 cells were also involved in the deregulation of cell shape, cell proliferation, transport, and cytoskeletal re-organization; all of which are well known features of malignant transformation. Previous studies have shown that the process of cellular transformation is closely linked to deregulated proliferation, leading to changes in cellular metabolism (51). Although the specific mechanisms of cellular transformation vary between viruses, the net overall effect is often very similar (52). Our results also showed that the DEGs induced by both HPV18E6 and HPV11E6 were involved in responses to cell signaling, cell communication, and signal transduction processes, confirming that HPV11E6 activated genes also influence processes known to induce cellular transformation. These activities reflect common pathways utilized by both HPV11E6 and HPV18E6 during their productive life cycles.

It is interesting that the metabolic process gene set was significantly enriched by both HPV11E6 and HPV18E6. It is known that tyrosine kinase 2 (Tyk2) complexes with E6 but that the Tyk2/E6 interaction is stronger with HPV18E6 than HPV11E6 (53). Our GO data showed that HPV11E6 affected a number of signaling pathways, including the JAK1, JAK2, and TYK2 interferon pathways. Since this was not the case for HPV18E6, the HPV11E6 effect is probably mediated *via* the JAK1 and TYK2 signaling pathways while the HPV18E6 induced effect is more likely *via* cellular transformation pathways. HPV18E6 did deregulate cancer pathways, CCR3 signaling in eosinophil and IL8 signaling and the COPD pathway. Deregulation of the cancer pathways was not unexpected since p53 is a known tumor suppressor that blocks aberrant cell growth and tumor development. The COPD pathway, on the other hand, is one of the interesting pathways that were significantly deregulated by HPV18E6 but not by HPV11E6. The COPD pathway is not directly related to HPV infection but is accompanied by upregulation of IL8, MMP1, and MMP9 genes that are implicated in cellular transformation. MMPs have been shown to be elevated in many cancers (54–59) and have been associated with persistent inflammation and cellular transformation (41–45). IL8 has been associated with metastasis in several cancers and its expression correlates with angiogenesis and tumorigenesis in numerous xenograft and orthotopic *in vivo* models (40). Other studies have also reported the overexpression of IL8 in human prostate cancer cells, with concomitant upregulation of MMP9 and collagenase activities (60). HPV18E6 expression may, thus, potentiate cellular transformation via the deregulation of MMP and IL8 genes as observed in our soft agar assay results.

Analysis of the pathways deregulated by the DEGs shared by both HPV11E6 and HPV18E6 (PCA data) showed PI3K/AKT signaling, telomerase signaling, and cell cycle signaling as the most significantly deregulated pathways. Several studies have shown that the PI3K/AKT pathway is overactive in many types of tumors and exhibits significant impact on glycolysis by stimulating expression of key glycolytic enzymes (38, 39). We propose that HPVE6-mediated cellular transformation *via* this pathway may lead to concomitant increases in glucose uptake and glycolytic flux, further strengthening the cellular transformation potential (52). The differential regulation of the cyclins and cell cycle regulation pathways is also consistent with other studies that showed that the high-risk HPVE6 proteins are capable of binding to cell cycle regulatory proteins and interfere with both the G1/S and G2/M cell cycle checkpoints more effectively than the low-risk HPVE6 proteins (61, 62). The reduced capacity of the low-risk HPV types to induce immortalization and transformation reside in their reduced ability to interact with the cell cycle components that result in the loss of multiple cell cycle checkpoints responsible for maintaining host genome fidelity (63, 64). Our findings are supported by previous siRNA knockdown studies that showed targeted inhibition of JAK1 and TYK2 resulted in an almost complete elimination of IL-17, IL-22, and TGFβ signaling functions that could be harnessed therapeutically to treat patients with psoriasis pathogenesis and cancer (33, 65–67).

Taken together, these findings support the notion that E6 from HPV types 11 and 18 may be involved in the initiation of cellular transformation, although with different efficiencies. This study focused on the early stages of HPVE6 infection and the ability of the low-risk HPV11E6 to induce cellular transformation. Furthermore, transient transfection of cells eliminated artifacts induced clonal selection of a subpopulation of cells as is the case in stably transfected cells employed in previous studies. We demonstrate that both HPV11E6 and HPV18E6 may initiate cellular transformation, although to a lesser extent by HPV11E6.

Data from this study will be useful in future studies geared toward functional analysis of potential biomarkers in cancer. Inhibition of some of the deregulated signaling pathways in will also allow us to understand the roles of the respective DEGs induced by HPVE6.

## Materials and Methods

### Maintenance of Cells in Culture

HaCaT Cells were cultured at 37°C in a humidified atmosphere of 5% CO_2_ in Dulbecco’s modified eagle’s medium (DMEM) supplemented with 10% heat inactivated fetal calf serum (FCS), 2 mM l-glutamine, 100 U/ml penicillin, and 100 µg/ml streptomycin (complete DMEM). The medium was changed every 2 days, and the cells were sub-cultured when 90% confluent, by dislodging the cells with 0.05% trypsin-EDTA. Once detached, the trypsin-EDTA was inactivated by the addition of 5 ml of complete DMEM. Cells were collected by centrifugation at 4,000 rpm for 5 min, resuspended in complete DMEM and re-plated at a ratio of 1:3. Cells were regularly checked for mycoplasma contamination.

### Preparation of Adeno-HPVE6 Constructs and Infection of HaCaT Cells

The assembly and production of recombinant adenovirus was accomplished as shown in Figure 1A. First, E6 from either HPV11 or HPV18 was cloned into pShuttle2. After amplification in *Escherichia coli* DH5α, the expression cassette was excised from pShuttle2 and inserted into the Adeno-X vector. Lastly, the recombinant Adeno-X DNA was transfected into human embryonic kidney (HEK) 293 cells for packaging into infectious adenovirus particles. Recombinant adenovirus was harvested by lysing the transfected HEK 293 cells and purified using the Adeno-X maxi purification kit (Clontech, USA). All safety procedures were observed as per the manufacturers instructions when working with the recombinant adenovirus. Confirmation of the HPVE6 constructs and viral titer was done as per the manufacturer’s instructions. HaCaT cells (2 × 10^5^) were plated in a 35-mm dish and incubated overnight until 70% confluency. The cells were washed once with PBS and infected at a multiplicity of infection (MOI) of 100 using a polycation (DEAE-Dextran). For infection, 1 ml of complete DMEM was mixed with the recombinant adenovirus and 1 µl of DEAE-dextran (7.5 µg/ml) was added drop-wise onto each well. Control cells were left uninfected. The plates were incubated at 37°C in a humidified atmosphere containing 5% CO_2_ for 1 h and centrifuged for 1 h at 2,000 rpm at 37°C. A fresh aliquot of DMEM/E6/DEAE-Dextran was added and the above step repeated. The cells were infected with the Adeno-HPVE6 for 48 h after which DNA, RNA, and protein were extracted and analyzed to confirmation the expression of HPV11E6 and HPV18E6 by qRT-PCR, western blotting, and confocal microscopy.

### RNA Preparation and qRT-PCR

Total RNA was extracted according to the procedure of Chomczynzki and Sacchi (68) using Trizol reagent (Bio-Rad, Munich). Complementary DNA (cDNA) was prepared from 2 µg of total RNA using the ImProm-II™ Reverse Transcription System (Promega, Madison) according to the manufacturers’ instructions. qRT-PCR assays were performed using SYBR^®^ FAST qPCR kit (KapaBiosystems) in a reaction volume of 20 µl. All amplifications were performed as follows: initial denaturation at 95°C for 5 min, followed by 45 cycles at 95°C for 30 s, and 60°C for 20 s, and 72°C for 5 s. Analyzed genes were amplified in triplicate using the Light Cycler 480II (Roche) and normalized to GAPDH expression in the same sample using the efficiency corrected comparative Ct model. A list of the primers used and their annealing temperatures is shown in Table 4.

**Table 4:** Primer sequences and annealing temperatures.

Primer	Sequence	Annealing temperature (°C)
BCLAF1	5′-CGCGTCGAAGGTAGCTCTAT-3′ 5′-TTGGAGCGACCCATTTCTTTT-3′	59
BRCA1	5′-GGCTATCCTCTCAGAGTGACATTTTA-3′ 5′-GCTTTATCAGGTTATGTTGCATGGT-3′	60
BUB1	5′-TCATTCATGGAGACATTAAAC-3′ 5′-CTGAGCATCTCAACACACTG-3′	56
CCL21	5′-CAAGCTTAGGCTGCTCCATC-3′ 5′-TCAGTCCTCTTGCAGCCTTT-3′	58
CCL26	5′- AACTCCGAAACAATTGTACTCAGCTG-3′ 5′-GTAACTCTGGGAGGAAACACCCTCTCC-3′	61
CYP24A1	5′-CAA ACCGTG GAAGGCTATC-3′ 5′-AGTCTTCCCCTTCCAGGATCA-3′	60
GADD45A	5′-GAAGTCCGCGGCCAGGACACAGTTCC-3′ 5′-GGTCCCCGCCGGGCTGTCACTCGG-3′	62
GAPDH	5′-GGCTCTCCAGAACATCATCC-3′ 5′-GCCTGCTTCACCACCTTC-3′	59
HPV11E6	5′-AAGATGCCTCCACGTCTGCAA-3′ 5′-CTTGCAGTTCTAGCAACAGGC-3′	60
HPV18E6	5′-CCAGAAACCGTTGAATCCAG-3′ 5′-GAGTCGTTCCTGTCGTGCTC-3′	59
IL8	5′-ACTGAGAGTGATTGAGAGTGGAC-3′ 5′-AACCCTCTGCACCCAGTTTTC-3′	59
JUN	5′-CCT TGAAAGCTCAGAACTCGG AG-3′ 5′-TGCTGCGTTAGCATG AGTTGG C-3′	61
MCM2	5′-GCTCTGGCCCTG TTTGGA-3′ 5′-GAAGATGGCACGGCTAGACAC T-3′	59
MCM6	5′-CTCTGAATGCCAGGACATCCA T-3′ 5′-CATTGCATTCATCCACCAGAA-3′	60
MMP1	5′-GGAGGGGATGCTCATTTTGATG-3′ 5′-TAGGGAAGCCAAAGGAGCTGT-3′	59
MMP9	5′-GGCCAACTACGACACCGACGAC-3′ 5′-CGCCGCCACGAGGAACAAAC-3′	61
MTTP	5′-GGACTT TTTGGATTTCAAAAGTGAC-3′ 5′-GGAGAAACGGTCATAATTGTG-3′	58
NOX1	5′-CACGCTGAGAAAGCCATTGGATCAC-3′ 5′-GGATGGAATAGGCTGGAGAGAACA-3′	61
P21	5′-ACCTCACCTGCTCTGCTGC-3′ 5′-ATTAGGGCTTCCTCTTGGAGA-3′	65
P53	5′-CATCATCACACTGGAAGACTCC-3′ 5′-CAGTGCTCGCTTAGTGCTCC-3′	63
PDK4	5′-CCCGCTGTCCATGAAGCAGC-3′ 5′-CCAATGTGGCTTGGGTTTCC-3′	61
STARD7	5′-GGTAATCAAGCTGGAGGTGATTG-3′ 5′-GAGTACATTGGATAAGGAAAATGGGT-3′	59
TP53INP1	5′-TGAACACATTTGCCTTGTGAA-3′ 5′-GGCAAAGTGCTGTGCTGTT-3′	60
TSHB	5′-GGCAAACTGTTTCTTCCCAA-3′ 5′-TCTGTGGCTTGGTGCAGTAG-3′	59
ZIC1	5′-AAACTGGTTAACCACATCCGC-3′ 5′-CTCAAACTCGCACTTGAAGG-3′	59

### Western Blot Analysis

HaCaT cells infected with HPVE6 constructs as described above were lysed in RIPA buffer (10 mM Tris–HCl pH 7.6, 10 mM NaCl, 3 mM MgCl2, and 1% (v/v) Nonidet P-40 containing 50 µg/ml each of pepstatin, leupeptin, and aprotinin). Total protein concentration was determined using the Bradford assay (Bio-Rad, Munich). Total cell lysates (50 µg protein) were separated by electrophoresis on 12% polyacrylamide/SDS gels under reducing conditions (50 mM β-mercaptoethanol). After electrophoresis proteins were transferred to nitrocellulose membranes and blocked in 5% fat-free milk in Tris-buffered saline (TBS) containing Tween-20. The membranes were incubated overnight at 4°C with one of the following primary antibodies: HPV18E6 (Arbor Vita Corporation, USA); p53 (Santa Cruz Biotechnology, USA); p21 (Santa Cruz Biotechnology, USA), or GAPDH (Santa Cruz Biotechnology, USA). After several washes in TBS–Tween buffer, the nitrocellulose membranes were incubated with the required horseradish peroxidase (HRP)-conjugated secondary antibodies and detected using LumiGLO substrate (KPL, Gaithersburg).

### Confocal Microscopy

A microscope cover slip was placed inside a 35-mm dish, and 10^5^ HaCaT cells were seeded onto the cover-slips. The cells were infected with HPVE6 constructs and incubated for 48 h at 37°C, 5% CO_2_, in a humidified atmosphere. The cells were rinsed twice in pre-warmed PBS and permeabilized in absolute methanol at −20°c for 5 min. The cells were fixed in 4% paraformaldehyde for 5 min at room temperature and washed three times in PBS for 10 min each. Cells were then incubated in blocking solution (1% BSA and 0.1% Triton X-100 in PBS) for 1 h at room temperature followed by incubation with the respective antibodies diluted 1:100 in blocking solution in a humidified chamber and incubated overnight at 4°C. The cells were washed thrice in PBS for 10 min each and incubated with the specific secondary antibodies diluted 1:500 in blocking solution. Thereafter, the cells were incubated for 90 min in a humidified chamber in the dark and washed thrice in PBS for 10 min each at room temperature. Slides were counterstained with DAPI Nuclear Stain (0.5 µg/ml in PBS) diluted 1:100 in blocking solution for 10 min and washed thrice in PBS for 10 min each at room temperature. The cells were mounted onto microscope slides with Mowiol containing n-propylgallate as anti-fading agent. The slides were stored in the dark at room temperature for the Mowiol to set and stored in the dark at 4°C until viewing. Slides were analyzed by confocal microscopy (Zeiss LSM 510 Meta with NLO).

### Soft Agar Assay

HaCaT cells infected with HPVE6 constructs were incubated at 37°C in 5% CO_2_ in a humidified atmosphere for 48 h. The media were removed and the cells washed with PBS, trypsinized, collected by centrifugation at 4,000 rpm for 5 min, and resuspended in 3 ml complete DMEM. A base agar was prepared by mixing 1% agarose (40°C) with 2× DMEM (20% FCS and 2% P/S). 1 ml of the mixture was added to each well of a 6 well plate and swirled to cover the entire surface. The plates were allowed to set at room temperature for 30 min before use. Once the base agar had set, a top agar base was prepared by melting 0.7% DNA agarose and cooling it to 37°C. 2.5 × 10^3^ cells in 0.1 ml of 2× DMEM, 20% FCS, and 0.7% DNA agar were added gently onto the warmed base agar and incubated at 37°C in 5% CO_2_ humidified incubator for 21 days with feeding of the cells with 1 ml complete DMEM one to two times per week. The cells were stained with 0.5 ml of 0.005% crystal violet for 1 h at 37°C in 5% CO_2_ humidified incubator. The crystal violet was removed and the colonies counted using an Olympus CKY41 light microscope at 100× magnification. Analysis of the colonies was done using the ImageJ software (69), with colonies larger than 150 µm in diameter being scored as positive. Graph shows mean number of colonies (±SE) obtained in three independent experiments, with each experiment performed in 6 replicate wells.

### Microarray Analysis

RNA samples were extracted as triplicate biological replicates and quantitated using the NanoDrop 2000/2000c Spectrophotometer (Thermo Scientific, IL, USA) followed by analysis in the Agilent Bioanalyzer nano assay to assess the integrity of the samples prior to downstream applications (Agilent Technologies, Germany). The prepared samples were hybridized overnight to the Affymetrix human gene ST 2.0 arrays, washed and stained using the GeneChip fluidics station 450 and scanned using the GeneChip^®^ scanner 3000 7G. An initial analysis was carried out to determine the quality of the data and the identification of any possible outliers. Raw intensity files were normalized using the RMA approach for affymetrix microarray chips (Affymetrix, USA). Analysis of variance (ANOVA) was used in identifying the DEGs with fold-change cutoff of ±2.0 with significance FDR of ≤0.05, using the Partek^®^ genomics suite™ 6.6 software. A web-based Venn diagram (70) was used to calculate and identify genes that were induced by HPVE6. GOTree machine (GOTM) (71) was used for GO analysis of the DEGs. The IPA system was used to identify the top most significantly regulated pathways from the Venn diagram list. The results were presented graphically based on scoring of the ratio of significant genes present in the canonical pathway to the total number of molecules in the canonical pathway. The *p*-value was calculated using a right-tailed Fisher Exact test and indicates the likelihood of the pathway association under the random model, and the threshold level was set at *p* = 0.05.

The microarray data have been deposited into the GEO database with the accession number GSE103546.

## Author Contributions

Conceived and designed the experiments: LM and MP. Performed the experiments: LM. Analyzed the data: LM, NT, and MIP. Contributed reagents/materials/analysis tools: NT and MP. Wrote the paper: LM and MP. All authors approved the final version of the manuscript.

## Conflict of Interest Statement

The authors declare that the research was conducted in the absence of any commercial or financial relationships that could be construed as a potential conflict of interest.
