# Supplementary material for: Delineation of the HPV11E6 and HPV18E6 Pathways in Initiating Cellular Transformation
Source: Front Oncol. 2017 Nov 1;7:258. doi: 10.3389/fonc.2017.00258 (PMC5672010; doi:10.3389/fonc.2017.00258)

**SUPPLEMENTARY FIGURE 1:** Partial image of NCBI HPVE6 Nucleotide sequence quality confirmation. A: Nucleotide sequence identity part of the HPV11E6 insert in adenoHPV11E6 using specific CMV promoter primers shows an excellent quality of the sequence using the chromas 2.01 software. B: Nucleotide sequence identity of part of HPV18E6 insert in adenoHPV18E6 using specific CMV promoter primers shows an excellent quality of the sequence using the chromas 2.01 software

**Supplementary Figure 1**


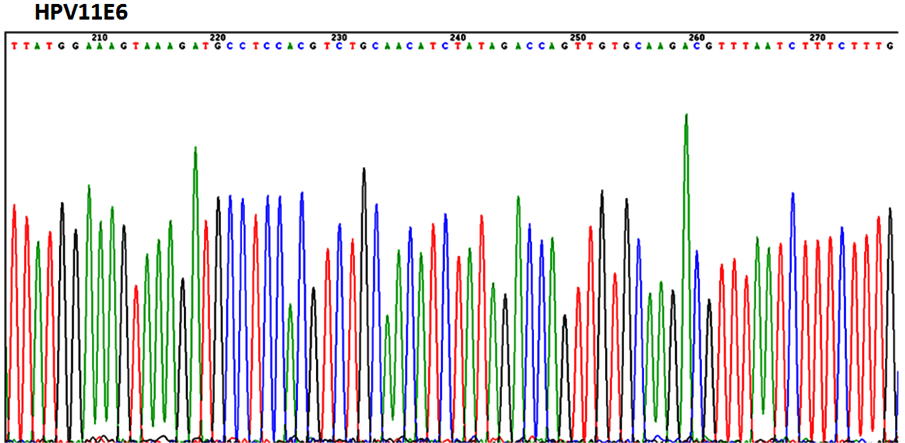


**B**


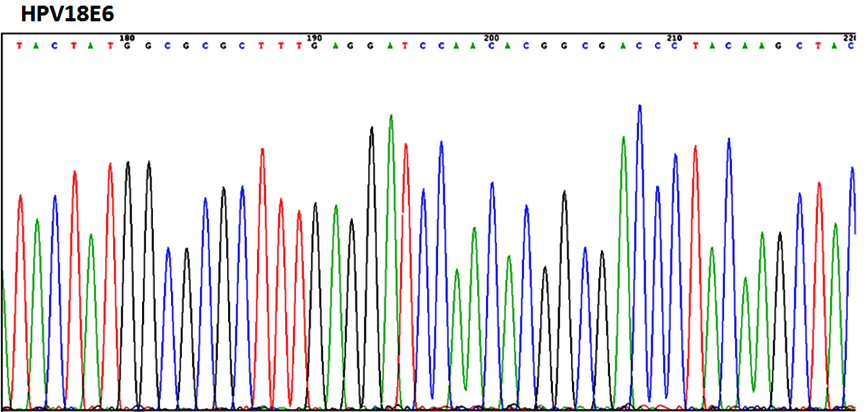

Supplement: Supplementary file 1 [file data_sheet_1.docx]
